# Ambler’s Journal of Dental Operations for 1848

**Published:** 1848-04

**Authors:** 


### AMBLER'S JOURNAL OF DENTAL OPERATIONS, FOR 1848.

This work, so necessary and important to the dentist, is gotten up in a neat style, and so arranged, that we have a separate plate and table for every day of the year, (Sundays excepted,) also for every two days a blank page for memoranda.

This Journal is particularly desirable, in that we so often plug teeth for those who have been operated upon before, by those whose fillings will not remain, and we often find the memory of our patients very little to be trusted, as to which teeth we had plugged. On these plates we can mark each tooth, and the place on each tooth where we have inserted a filling, and by reference, at once remove all doubt as it regards whose plugs have dropped out.

We should be glad to see all our dentists use the above work, or one of the kind, for their account book, and thus throw the blame of bad work where it always should be.

We hope the work may meet with extensive sale.—CIN. ED.

